# Cannabis Use and Risk of Periodontitis and Adverse Oral Health Outcomes: A Systematic Literature Review

**DOI:** 10.7759/cureus.114734

**Published:** 2026-08-18

**Authors:** Joanne Son, Brianna Bramswig, Connor Orlowski, Anastasiya Shor

**Affiliations:** 1 Ambulatory Care, Touro College of Pharmacy, New York, USA; 2 Dental Medicine, Touro College of Dental Medicine, Hawthorne, USA; 3 Pharmacy, Touro College of Pharmacy, New York, USA; 4 Drug Information, Touro College of Pharmacy, New York, USA

**Keywords:** anesthesia, cannabis, caries, dental, marijuana, oral health, periodontitis, xerostomia

## Abstract

Cannabis is widely consumed globally. Common methods of consumption include smoking, vaping, and consuming edibles. Despite its increasing use, the impact of cannabis consumption on the treatment and management of oral health disorders remains poorly understood. Cannabis has been associated with adverse oral effects such as periodontitis, xerostomia, caries, and gingivitis. These adverse oral effects complicate dental care. A systematic literature review was conducted, searching PubMed, EMBASE, and Cochrane databases from inception to May 31, 2024, to evaluate the effects of cannabis use on oral health, including its association with periodontitis and other adverse outcomes relevant to the treatment and management of oral health disorders among current or former adult users compared with never users. Eligible studies included randomized controlled trials (RCTs), quasi-RCTs, cohort studies, case-control studies, and cross-sectional surveys involving cannabis users and never users with diagnosed dental disorders; articles including synthetic cannabinoids were excluded; relevant articles were restricted to English-language studies on human subjects. The review protocol was registered with PROSPERO (CRD42024558418). Abstract and full-text reviews were conducted by two independent reviewers, with conflicts resolved by a third adjudicator. Crowe Critical Appraisal Tool (CCAT) Version 1.4 was used to assess the risk of bias within the included articles. The included studies were thematically analyzed and presented in descriptive tables.

A total of 6,535 records were identified; after abstract and full-text review, 23 articles were included and underwent data extraction. The most prevalent condition was periodontitis, consisting of 11 studies, of which nine identified a negative relationship between cannabis use and periodontitis. Overall, cannabis use appears to be associated with an increased risk of adverse health outcomes like periodontitis, highlighting the need for patient screening. Other studies addressed health disorders such as cancer, dental caries, xerostomia, and altered anesthesia requirements. The interpretation of study results was limited by variability in study methodologies and reported outcomes. This work was supported by the Touro College of Pharmacy Student Summer Research Fellowship.

## Introduction and background

Used by 244 million people in 2023, cannabis is the fourth most-commonly used substance behind alcohol, tobacco products, and nicotine vaping in the United States [1,2]. As of June 2024, recreational cannabis is legal in 24 states, two territories, and the District of Columbia within the United States [3]. Despite ongoing discussions by the Drug Enforcement Agency to reschedule this product, cannabis currently remains a Schedule I substance under the federal Controlled Substances Act, and its widespread use may occur without clinical oversight [4]. The highest rates of use occur among adults aged 18-25 (36.5% or 12.4 million people), followed by adults aged 26 or older (20.8% or 46.5 million people) [2], with use more prevalent among men and people with annual household incomes below $20,000 [5].

Cannabis consumption methods include smoking, vaping, and ingestion of edibles. Recent trends suggest a shift from smoking to vaping, particularly among younger adults [6]. The plant contains several active cannabinoids, primarily delta-9-tetrahydrocannabinol (THC), cannabidiol (CBD), delta-8-THC, and cannabinol. These compounds vary in concentration depending on the cannabis strain, and the ratio of THC to CBD influences both the pharmacologic and psychoactive profiles [7]. Inhaled cannabis formulations tend to produce higher peak plasma concentrations of THC than vaped products, although variability exists based on individual metabolism and product formulation [8]. 

While the systemic effects of cannabis have been well documented, its impact on oral health remains less clear. Some studies suggest associations between cannabis use and conditions such as periodontitis, xerostomia, dental caries, gingivitis, and oral mucosal disease. However, most evidence is limited to small or poorly controlled studies, often failing to consider mode of administration, frequency of use, or concurrent use with tobacco [9-13]. As a result, clinicians lack reliable, evidence-based guidance for managing cannabis users in dental settings. 

The growing prevalence of cannabis use, particularly among younger adults [5], creates a need for greater clinical awareness of its potential effects on oral health. Currently, consolidated literature in this space is lacking. This work is intended to aggregate sparse findings and support dental professionals in recognizing, assessing, and managing the oral health needs of patients who use cannabis. The purpose of this systematic literature review (SLR) is to evaluate the effects of cannabis consumption on oral health outcomes and their implications for dental treatment and management. Specifically, this SLR aims to assess whether adult users of cannabis (current or former) are at higher risk of periodontitis and other adverse oral health effects compared with never users. Preliminary findings were previously presented as a meeting abstract at the 59th American Society of Health-System Pharmacists (ASHP) Midyear Clinical Meeting and Exhibition, New Orleans, LA, on December 8-12, 2024; an encore presentation was delivered at the New York State Council of Health-System Pharmacists (NYSCHP) Annual Assembly, Saratoga Springs, NY, on April 3-6, 2025.

## Review

Methods 

This SLR was executed in accordance with the Preferred Reporting Items for Systematic Reviews and Meta-Analyses (PRISMA) 2020 statement [14]. The completed PRISMA checklists are available upon request. Included studies described all current literature related to the effects of cannabis on oral health outcomes and their implications for the treatment and management of oral health disorders, comparing current or former users with never users. 

Search strategy 

A comprehensive search strategy was developed and applied by AS and JS to ensure that all relevant articles were captured from PubMed, EMBASE, and Cochrane. The PICOS (Population, Intervention, Comparator, Outcome, Study Design) process was utilized to explore these databases as described in Table 1 and Table 2. Included studies compared outcomes of ever users of cannabis, defined as current, recent, or former users, with never users. The limitations applied to the search strategy included articles written in the English language, studies conducted on human subjects, and articles available from database inception to May 31, 2024 (Table 3). 

**Table 1:** Inclusion Criteria

Element	Inclusion criteria
Participants	Patients receiving treatment for dental disease, dental professional providing treatment for dental disease
Intervention	Cannabis, marijuana
Comparators (Control)	Never user of cannabis or marijuana
Outcomes	Treatment and management of dental diseases influenced by cannabis
Study design	Analytical trials (randomized, clinical trial, controlled trial intervention, observational)

**Table 2:** Exclusion Criteria

Element	Exclusion criteria
Participants	Epilepsy, multiple sclerosis, ulcerative colitis, Crohn’s disease, cannabis use disorder (unless in context of oral treatment), cancer pain (unless in context of oral treatment), alcohol use disorder
Intervention	Synthetic cannabinoid agonists, cannabinoid antagonists, opiates, cannabidiol
Comparators (Control)	Not applicable
Outcomes	Changes unrelated to management or treatment of dental disease
Study design	Descriptive studies, i.e., case studies, reviews; systematic literature reviews; articles published before 1980

**Table 3:** Search Strategy MeSH, Medical Subject Headings.

Source (Database/Register/Website)	Platform	Full search strategy	Limits/Filters applied	Results	Date of search
PubMed	PubMed (MEDLINE)	("cannabis"[MeSH Terms] OR "cannabis"[All Fields] OR "cannabi"[All Fields] OR "cannabis s"[All Fields] OR ("cannabis"[MeSH Terms] OR "cannabis"[All Fields] OR "marijuana"[All Fields] OR "marijuana s"[All Fields]) OR ("cannabinoids"[MeSH Terms] OR "cannabinoids"[All Fields] OR "cannabinoid"[All Fields])) AND ((("mouth"[MeSH Terms] OR "mouth"[All Fields] OR "oral"[All Fields]) AND ("therapeutics"[MeSH Terms] OR "therapeutics"[All Fields] OR "treatments"[All Fields] OR "therapy"[Subheading] OR "therapy"[All Fields] OR "treatment"[All Fields] OR "treatment s"[All Fields])) OR ("dentistry"[MeSH Terms] OR "dentistry"[All Fields] OR "dentistry s"[All Fields]) OR ("dental health services"[MeSH Terms] OR ("dental"[All Fields] AND "health"[All Fields] AND "services"[All Fields]) OR "dental health services"[All Fields] OR "dental"[All Fields] OR "dentally"[All Fields] OR "dentals"[All Fields]))	English; Humans	1,800 (after filters); 2,786 initial	May 31, 2024
Embase	Embase (Elsevier)	#1 'cannabis'/exp OR cannabis OR 'marijuana'/exp OR marijuana OR 'cannabinoid'/exp OR cannabinoid; #2 oral AND treatment OR dentistry OR dental ; #3 #1 AND #2	English; Humans	4,694 (after filters); 6,013 initial	May 31, 2024
Cochrane Library	Cochrane Database of Systematic Reviews	Cannabis OR marijuana OR cannabinoid in title, abstract, keyword		40 reviews	May 31, 2024

Screening process 

Following review planning, the title and protocol were registered and accepted by PROSPERO, an international database of prospectively registered systematic reviews in health and social care (CRD42024558418). The platform, PICO Portal, was employed to conduct the review. 

PICO Portal is an online platform that utilizes machine learning and natural language processing algorithms for SLR. Data extraction was carried out independently by two reviewers (BB and CO) using a standardized form; disagreements between the reviewers on article eligibility were reviewed by the principal reviewers (AS and JS) acting as adjudicators. All eligible articles from the abstract review process underwent full-text review in the same manner, with blinded reviewers and adjudicators. 

At no point was artificial intelligence (AI) used to execute any part of the screening or analysis of articles reviewed. PICO Portal implemented a beta form of AI in the abstract reviewing process. Here, AI served as an aid by highlighting potentially relevant terms (e.g., cannabis) in various colors, and non-relevant terms (e.g., cystic fibrosis) in red. AI highlights could be used at various strengths, including "most," "some," "keywords," and "none.” This tool could be changed at any time during the review process at the reviewer's individual discretion. 

Data analysis 

After the initial full-text review, the included articles underwent data extraction and qualitative analysis. The extraction categories included country/setting, study design, population studied, sample size, intervention type, duration, outcomes, type of outcome as it relates to cannabis, baseline characteristics as they relate to cannabis, results as they relate to cannabis, conclusion, strengths, and limitations. Due to heterogeneity of the data, a meta-analysis could not be conducted. A summary of the included studies is presented, with key themes highlighted in the Results section. 

Quality assessment 

To determine the overall risk of bias, the Crowe Critical Appraisal Tool (CCAT) version 1.4 was utilized to assess each article [15]. With this tool, each article was assessed independently by two reviewers (BB and CO) in accordance with the following categories: preliminaries, introduction, design, sampling, data collection, ethical matters, results, discussion. Separately, each reviewer rated each section from 0 to 5 for a total score out of 40 points. After completion of the reviews, the two scores were averaged. If there was ≥20% difference in the score for each article between the two reviewers, then the article in question was further reviewed by adjudicators (AS and JS). The score determined by the adjudicators was then averaged with the next closest score of one of the reviewers (BB or CO). 

Results 

**Figure 1:**
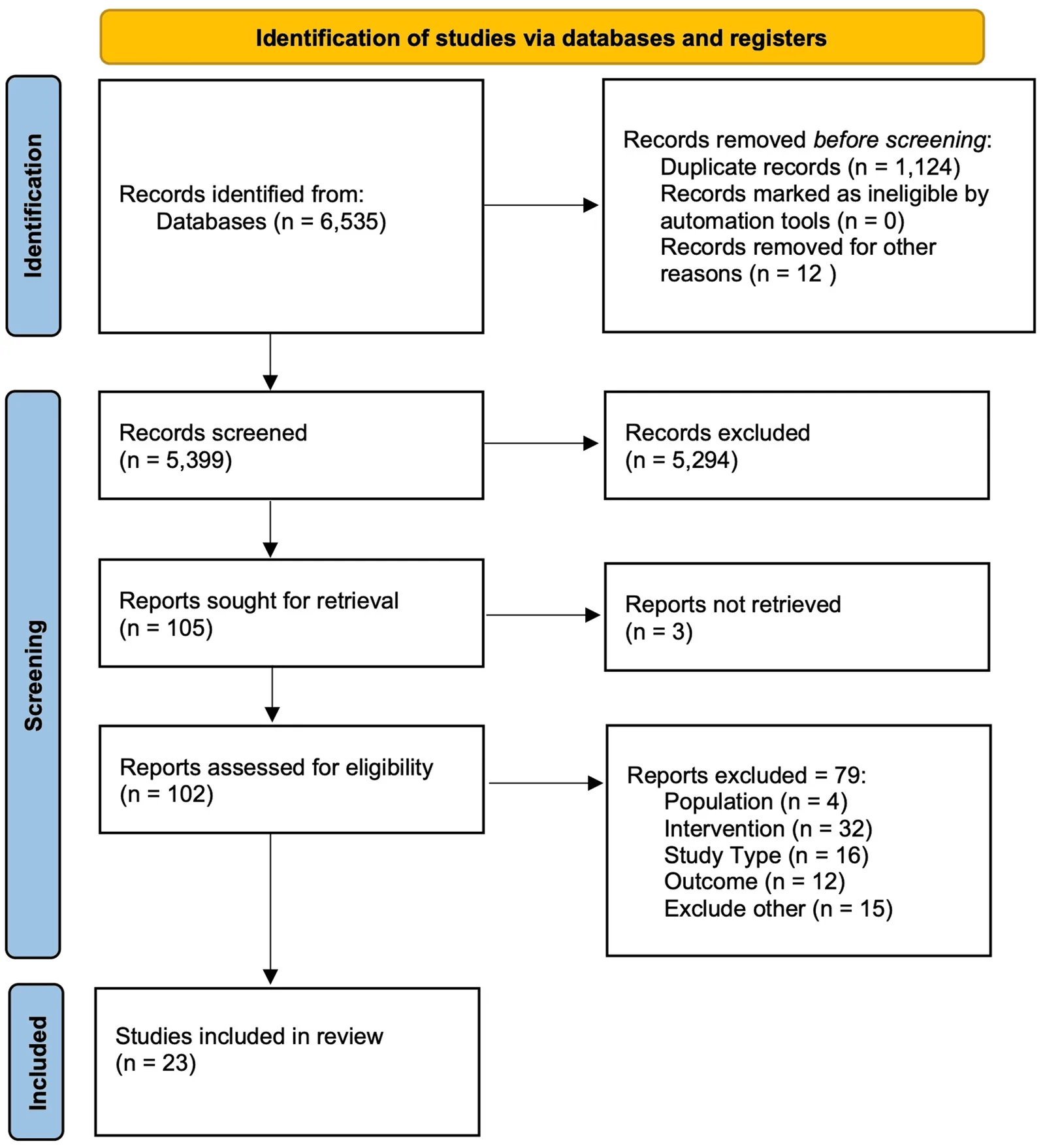
PRISMA Diagram

A total of 6,535 records were identified through Embase (4,378 records), PubMed (2,101 records), Cochrane (40 records), and a bibliographic review (16 records), of which 1,136 were removed before screening as duplicates or supplemental records. After abstract review, 105 full-text records were sought for retrieval. Of these, 102 records were retrievable and assessed for eligibility. Full-text review excluded 79 records for the following reasons: ineligible population (four records), interventions (32 records), study type (16 records), outcomes (12 records), and other reasons (15 records). The remaining 23 records underwent data extraction (Figure 1) [16-38]. A summary of included studies is outlined in Table 4.

**Table 4:** Included Studies CCAT, Crowe Critical Appraisal Tool.

First author et al., year	Title	Risk-of-bias score (CCAT v1.4)
Baumeister et al., 2022 [16]	Cannabis Use and the Risk of Periodontitis: A Two-Sample Mendelian Randomization Study	85%
Chaffee et al., 2023 [36]	E-cigarette, Cannabis and Combustible Tobacco Use: Associations With Xerostomia Among California Adolescents	88%
Chatzopoulos et al., 2023 [17]	Association Between Periodontitis Extent, Severity, and Progression Rate With Systemic Diseases and Smoking: A Retrospective Study	85%
Ditmyer et al., 2013 [32]	The Effect of Tobacco and Marijuana Use on Dental Health Status in Nevada Adolescents: A Trend Analysis	85%
Gillison et al., 2008 [27]	Distinct Risk Factor Profiles for Human Papillomavirus Type 16-Positive and Human Papillomavirus Type 16-Negative Head and Neck Cancers	95%
Helmi et al., 2024 [33]	Oral Health Status Among Recreational Cannabis (Marijuana and Hashish) Users in the USA: A NHANES-Based Cross-sectional Study	93%
Jamieson et al., 2010 [18]	Substance Use and Periodontal Disease Among Australian Aboriginal Young Adults	90%
Javed et al., 2020 [19]	Periodontal Conditions and Whole Salivary IL-17A And -23 Levels Among Young Adult Cannabis Sativa (Marijuana)-Smokers, Heavy Cigarette-Smokers and Non-smokers	80%
Kerouad et al., 2021 [28]	Tumor Site Related Factors in Patients With Upper Aerodigestive Tract Cancer in Morocco	75%
Le et al., 2022 [20]	Associations Between Oral Health and Cannabis Use Among Adolescents and Young Adults: Implications for Orthodontists	85%
Llewellyn et al., 2004 [29]	An Analysis of Risk Factors for Oral Cancer in Young People: A Case-Control Study	95%
Lopez et al., 2009 [21]	Cannabis Use and Destructive Periodontal Diseases Among Adolescents	88%
Meier et al., 2016 [22]	Associations Between Cannabis Use and Physical Health Problems in Early Midlife: A Longitudinal Comparison of Persistent Cannabis vs Tobacco Users	90%
Moran et al., 2022 [37]	Local Anesthetic Efficacy in Marijuana Users and Nonusers: A Pilot Study	83%
Ripperger et al., 2023 [38]	Cannabis Users Require More Anesthetic Agents for General Anesthesia in Ambulatory Oral and Maxillofacial Surgery Procedures	83%
Samman et al., 2024 [34]	The Effect of Marijuana-Smoking on Dental Caries Experience	80%
Schulz-Katterbach et al., 2009 [35]	Cannabis and Caries - Does Regular Cannabis Use Increase the Risk of Caries in Cigarette Smokers	80%
Shah et al., 2018 [30]	Association of Life Style Factors in Patients Having Head and Neck Carcinomas Visiting Dental Hospitals	68%
Shariff et al., 2017 [23]	Relationship Between Frequent Recreational Cannabis (Marijuana and Hashish) Use and Periodontitis in Adults in the United States: National Health and Nutrition Examination Survey 2011 to 2012	88%
Shewale et al., 2021 [31]	Independent Association of Marijuana Use and Poor Oral Hygiene With HPV-Negative but Not HPV-Positive Head and Neck Squamous Cell Carcinomas	93%
Thomson et al., 2008 [24]	Cannabis Smoking and Periodontal Disease Among Young Adults	88%
Thomson et al., 2013 [25]	The Natural History of Periodontal Attachment Loss During the Third and Fourth Decades of Life	93%
Zeng et al., 2014 [26]	Reexamining the Association Between Smoking and Periodontitis in the Dunedin Study With an Enhanced Analytical Approach	93%

Two rounds of blinded analyses were conducted, with discrepancies in scores greater than 20% adjudicated by a third reviewer. The quality of included articles was assessed using the CCAT Version 1.4, which assigns a maximum score of 40 points (100%). The included studies achieved an average CCAT score of 34.4 out of 40 (86%). 

Periodontitis 

An association between cannabis use and periodontitis was described in 11 of the 23 studies reviewed [16-26]. Of the 11 studies included, five were cross-sectional [17-21], four were cohort [22,24-26], one was observational [23], and one was a two-sample Mendelian randomization study [16]. The majority of studies reported a negative association between cannabis and periodontal health, with cannabis linked to worse clinical and radiographic indicators of periodontitis [18,19,22,23,25,26]. Three studies found no association between cannabis use and risk or severity of periodontal disease [16,17,21]. 

Nine of the 11 studies identified a negative relationship between cannabis use and dental health, specifically periodontitis [17-20,22-26]. Frequent cannabis use was commonly linked to more severe signs of periodontal disease such as deeper gum pockets, more attachment loss, and higher rates of tooth loss. These findings were generally seen in people who used cannabis weekly or daily over extended periods. Some studies found that cannabis users showed similar levels of gum disease as cigarette smokers, and a few even found that the impact of cannabis might be worse when used in combination with poor oral hygiene or other substances such as cocaine or heroin.  

Ten studies evaluated the relationship between periodontitis and cannabis use among ever users and never users [17-26]. Most of these found that cannabis users had worse periodontal outcomes, including deeper probing depths and greater clinical attachment loss. While not all studies quantified the magnitude of effect, the overall trend supported a negative association between cannabis use and periodontal health when comparing ever users to never users. 

Several studies also raised concerns about long-term effects. People who regularly used cannabis over many years tended to show worse gum health than non-users. However, a few reports found that occasional or infrequent cannabis use was not clearly linked to poorer dental outcomes. In addition, some studies identified that behavioral factors, such as poor oral hygiene and higher consumption of sugary beverages and sweets, may indirectly increase periodontitis risk [19,20,22]. Le A et al. reported that 46.7% of cannabis users frequently consumed sugary beverages or snacks compared to 26.3% of non-users (adjusted prevalence ratio 1.26; 95% CI 1.07-1.48) [20]. As well, Javed F et al. reported that 20% of marijuana smokers brushed twice a day compared to 31.2% of non-smokers with periodontitis [19]. 

Three studies did not find any strong connection between cannabis use and periodontal disease [16,17,21]. One large genetic study found no evidence of a direct biological link in cannabis users, concluding that a predisposition to cannabis use is unlikely to be associated with periodontitis (OR 1.05; 95% CI 0.93-1.19; p = 0.411) [16]. Two additional studies involving self-reported cannabis use, particularly in younger or adolescent populations, found no significant link to tooth loss or gum issues. Chatzopoulos and colleagues found no significant difference in implant failure between marijuana users and non-users (OR 1.471; 95% CI 0.815-2.656; p = 0.200) [17]. López and Baelum described periodontal disease by the presence of necrotizing ulcerative gingival (NUG) lesions or clinical attachment loss ≥3 mm. For regular users of cannabis, there was no significant difference in occurrence of NUG lesions (OR 0.85; 95% CI 0.5-1.4) nor clinical attachment loss ≥3 mm (OR 0.77; 95% CI 0.4-1.4) [21]. 

Other oral health disorders 

Six studies described the association of cannabis use and cancer [17,27-31]. It was found that cannabis use is connected to human papillomavirus (HPV)-related cancers [27,28,31]. One study showed an association between a high number of marijuana-hit years (smoking an average of one hit of marijuana per day for one year) and HPV-negative oral squamous cell carcinoma; however, after adjusting for tobacco and alcohol use, there was no association (lifetime hit-years ≥10, <50 OR 1.30, 95% CI 0.61-2.79; hit-years ≥50 OR 1.65, 95% CI 0.94-2.90) [31]. Two studies associated cannabis use with HPV-16-positive head and neck squamous cell carcinomas (HNSCC) (current users adjusted OR 4.7; 95% CI 1.3-17) [27] and laryngeal cancer (p = 0.042) [28]. A weaker association between marijuana smoking and HPV-16-negative HNSCC was found. In contrast, three studies showed no association between cannabis and cancers [17,29,30]. 

Six studies discussed the burden of dental caries in cannabis users versus non-users, of which three concluded that there was no difference between the two groups [19,20,32-35]. Specifically, the literature described an association between cannabis use and a higher occurrence of missing teeth or decayed surfaces. Conversely, three studies showed no direct link between cannabis use and dental caries, missing teeth, or the DMFT index (Decayed, Missing, and Filled Teeth) [20,33,34]. Some data suggest that cannabis users are more likely to consume sugary beverages (Schulz-Katterbach M et al. [35], p < 0.0078; Samman M et al. [34], p < 0.001) or engage in behaviors that contribute to cariogenic environments [20,34,35]. 

Ditmyer M et al. compared DMFT indices for marijuana users and non-users over the course of eight years [32]. For marijuana users, the mean indices increased from 3.11 in year 1 to 4.00 in year 8 (p < 0.05). This difference was not observed among non-users, where the average DMFT indices were 2.15 in year 1, then 2.99 in year 8. Samman M et al. directly compared average DMFT indices between nonsmokers (8.72), previous marijuana smokers (9.49), and current marijuana smokers (9.87). There was an increase in average DMFT indices in marijuana smokers compared to nonsmokers (p < 0.0001). However, the authors found no difference after controlling for confounders, including sociodemographic factors [34]. 

The association of xerostomia and cannabis use was described by three studies [19,35,36]. A strong association between frequent cannabis use and the subjective experience of xerostomia in adolescents was identified with frequent cannabis use (OR 3.17; 95% CI 1.47-6.82) [36]. However, no differences in stimulant salivary flow were reported [19,35]. 

Two articles discussed anesthesia requirements relative to cannabis use [37,38]. When undergoing deep sedation or general anesthesia, higher amounts of propofol (users 152.5 mg ± 101.8; non-users 117.9 mg ± 71.3; p = 0.004), midazolam (users 5.1 mg ± 1.5; non-users 4.7 mg ± 1.0; p = 0.01), ketamine (users 46.0 mg ± 16.9; non-users 40.1 mg ± 15.7; p = 0.01), and fentanyl (users 88.6 µg ± 32.8; non-users µg ± 26.3; p = 0.02) were required in cannabis users compared to non-users [38]. There was no difference in onset and success of local anesthetics (lidocaine with epinephrine) observed in cannabis users compared to non-users [37]. Onset within 10 minutes and lasting for at least 15 minutes were achieved in 88% of non-users and 61% of cannabis users (p = 0.73). 

Discussion 

Evidence regarding the effects of cannabis on oral health remains somewhat inconsistent and methodologically limited. The risks of periodontitis and other adverse oral health effects in current or former users of cannabis compared with never users have not been previously described in detail in the literature. Of the relevant literature identified in this review, the majority discussed periodontal disease and cannabis use [16-26]. Other notable adverse oral health effects include cancer [17,27-31], dental caries [19,20,32-35], xerostomia [19,35,36], and increased anesthesia requirements [37,38]. 

Regarding periodontal disease, the studies identified yielded mixed results. Some showed an increased risk of periodontal disease with cannabis use, while others showed no association. Eleven of the 23 included studies addressed this association [16-26]. Most of these studies suggest a positive association, particularly for frequent (daily or weekly) and long-term users, who exhibited deeper gum pockets and higher rates of attachment loss [17-20,22-26]. However, findings are not universal; for instance, a two-sample Mendelian randomization study found no direct biological link, and some research on younger populations showed no increase in gum issues [16]. This suggests that while cannabis use may be associated with periodontal disease, it may also be influenced by duration of use and individual susceptibility. Periodontal risk assessments should be routinely performed in patients who use cannabis, particularly in frequent or long-term users. 

While users subjectively report high rates of xerostomia (dry mouth), objective measurements of salivary flow have been inconclusive, suggesting a potential sensory or qualitative change in saliva rather than a purely quantitative one. The increased risk of dental caries with cannabis use can be further attributed indirectly to behavioral factors, such as sugary beverage consumption and poor oral health [20,34,35]. The "munchies" (frequent consumption of sugary beverages and snacks) appears to be a primary driver; one study noted that 46.7% of cannabis users frequently consumed sugary snacks compared to 26.3% of non-users [20]. Educating patients who use cannabis on proper dental hygiene, such as brushing and flossing regularly, may be a beneficial management strategy to improve oral health in this population. 

The evidence for cancer was mixed, which made it challenging to correlate the relationship between cancer and cannabis use [17,27-31]. Regarding head and neck cancer, there was evidence of increased odds of HPV-positive cancers [27] and no association of HPV-negative cancers [28] and cannabis use. These findings are not conclusive; however, they serve a hypothesis-generating function for further studies. Users undergoing deep sedation or general anesthesia required higher doses of propofol, midazolam, ketamine, and fentanyl compared to non-users [38]. Interestingly, this increased requirement did not extend to local anesthetics like lidocaine, where onset and success rates remained comparable between groups [37]. Thus, providers should take cannabis use into consideration when planning general anesthesia or deep sedation. 

The search strategy was intentionally broad to capture literature addressing both therapeutic and management-related aspects of oral health conditions in the context of cannabis use, which is consistent with the original objective. During screening and data extraction, it became evident that many of the available studies focused on associations between cannabis consumption and oral health outcomes like periodontitis, xerostomia, and dental caries rather than interventional treatment or management strategies. There were no changes made to the search strategy, but the synthesis was refined with greater focus on risk profiles and clinical implications for oral health management among users of cannabis. This represents a data-driven refinement in emphasis to better represent the identified evidence. 

This study highlights the common oral health trends observed in cannabis users. This may assist dental health professionals with counseling points and treatment considerations when working with this population. Methodologic strengths include prospective protocol registration with PROSPERO and reporting in accordance with PRISMA guidelines [14]. Furthermore, the included evidence was of high overall quality; all but two studies scored 80% or higher on the CCAT [15]. 

Initially, we considered using RoB-2 and ROBINS-I to assess overall risk of bias and methodological quality; the final set of studies comprised a very diverse range of study designs [39,40]. Therefore, CCAT was selected as a more suitable tool due to its ability to consistently evaluate methodological quality across heterogeneous study designs and standardize the evaluative framework [15]. 

Multiple limitations were identified in the relevant literature, including limited availability of longitudinal data and the potential for publication bias and underreporting. Our findings should be interpreted with caution as we were unable to perform a meta-analysis due to considerable clinical and methodological heterogeneity across the studies. The definition of cannabis users among the studies was inconsistent, making meaningful comparisons a challenge. Tobacco and alcohol use were found to be confounding factors in several studies. Use often co-occurred with cannabis and was not easily adjusted for. This ultimately complicated causal interpretations. In addition, the description of dose, frequency, mode, and duration of cannabis use varied widely across studies. Most studies relied on cross-sectional, subjective self-reported cannabis use. A major challenge in interpreting the current literature is the lack of standardized cannabis exposure assessment. This limits the ability to determine whether the adverse oral health outcomes are associated with specific patterns of use and cumulative exposure, or if specific types of consumption carry greater risk. This SLR covers available evidence through May 31, 2024. The interval between the search cutoff and manuscript completion reflects the time required for screening, data extraction, analysis, interpretation, and manuscript preparation.

## Conclusions

Cannabis use has been associated with adverse oral health outcomes, with the most consistent findings observed linking cannabis use to periodontal disease. Additional associations have been reported for xerostomia, dental caries, certain cancers, and potentially increased anesthetic requirements; however, these findings are based largely on observational studies and therefore should not be interpreted as causal. Given these potential risks, dental professionals should routinely assess and document cannabis use, including frequency, duration, route, and last-use date, to determine the volume of use and the likelihood of the effect. However, the current body of evidence is limited, highlighting the need for high-quality, longitudinal studies that use standardized measures of cannabis exposure to better define these associations. 
